# A Sensitive and Rapid Wastewater Test for SARS-COV-2 and Its Use for the Early Detection of a Cluster of Cases in a Remote Community

**DOI:** 10.1128/aem.01740-21

**Published:** 2022-03-08

**Authors:** Jade Daigle, Kathleen Racher, Justin Hazenberg, Allan Yeoman, Heather Hannah, Diep Duong, Umar Mohammed, Dave Spreitzer, Branden S. J. Gregorchuk, Breanne M. Head, Adrienne F. A. Meyers, Paul A. Sandstrom, Anil Nichani, James I. Brooks, Michael R. Mulvey, Chand S. Mangat, Michael G. Becker

**Affiliations:** a Wastewater Surveillance Unit, National Microbiology Laboratory, Public Health Agency of Canada, Winnipeg, Manitoba, Canada; b Taiga Environmental Laboratory, Department of Environmental and Natural Resources, Government of the Northwest Territories, Yellowknife, Northwest Territories, Canada; c Department of Municipal and Community Affairs, Government of the Northwest Territories, Yellowknife, Northwest Territories, Canada; d Department of Health and Social Services, Government of the Northwest Territories, Yellowknife, Northwest Territories, Canada; e JC Wilt Infectious Diseases Research Centre, National Microbiology Laboratory, Public Health Agency of Canada, Winnipeg, Manitoba, Canada; f Department of Medical Microbiology and Infectious Diseases, University of Manitoba, Winnipeg, Manitoba, Canada; g One Health Division, National Microbiology Laboratory, Public Health Agency of Canada, Guelph, Ontario, Canada; h Antimicrobial Resistance Division, Centre for Communicable Diseases and Infection Control, Public Health Agency of Canada, Ottawa, Ontario, Canada; i Division of Infectious Diseases, University of Ottawa, Ottawa, Ontario, Canada; j Antimicrobial Resistance and Nosocomial Infections, National Microbiology Laboratory, Public Health Agency of Canada, Winnipeg, Manitoba, Canada; k Department of Microbiology, University of Manitoba, Winnipeg, Manitoba, Canada; University of Nebraska-Lincoln

**Keywords:** COVID-19, GeneXpert, remote, SARS-CoV-2, wastewater rapid testing, wastewater surveillance, wastewater-based epidemiology

## Abstract

Throughout the coronavirus disease 2019 (COVID-19) pandemic, wastewater surveillance has been used to monitor trends in severe acute respiratory syndrome coronavirus 2 (SARS-CoV-2) prevalence in the community. A major challenge in establishing wastewater surveillance programs, especially in remote areas, is the need for a well-equipped laboratory for sample analysis. Currently, no options exist for rapid, sensitive, mobile, and easy-to-use wastewater tests for SARS-CoV-2. The performance of the GeneXpert system, which offers cartridge-based, rapid molecular clinical testing for SARS-CoV-2 in a portable platform, was evaluated using wastewater as the input. The GeneXpert demonstrated a SARS-CoV-2 limit of detection in wastewater below 32 copies/mL with a sample processing time of less than an hour. Using wastewater samples collected from multiple sites across Canada during February and March 2021, a high overall agreement (97.8%) was observed between the GeneXpert assay and laboratory-developed tests regarding the presence or absence of SARS-CoV-2. Additionally, with the use of centrifugal filters, the detection threshold of the GeneXpert system was improved to <10 copies/mL in wastewater. Finally, to support on-site wastewater surveillance, GeneXpert testing was implemented in Yellowknife, a remote community in Northern Canada, where its use successfully alerted public health authorities to undetected transmission of COVID-19. The identification of SARS-CoV-2 in wastewater triggered clinical testing of recent travelers and identification of new COVID-19 cases/clusters. Taken together, these results suggest that GeneXpert is a viable option for surveillance of SARS-CoV-2 in wastewater in locations that do not have access to established testing laboratories.

**IMPORTANCE** Wastewater-based surveillance is a powerful tool that provides an unbiased measure of COVID-19 prevalence in a community. This work describes a sensitive wastewater rapid test for SARS-CoV-2 based on a widely distributed technology, the GeneXpert. The advantages of an easy-to-use wastewater test for SARS-CoV-2 are clear: it supports surveillance in remote communities, improves access to testing, and provides faster results allowing for an immediate public health response. The application of wastewater rapid testing in a remote community facilitated the detection of a COVID-19 cluster and triggered public health action, clearly demonstrating the utility of this technology. Wastewater surveillance will become increasingly important in the postvaccination pandemic landscape as individuals with asymptomatic/mild infections continue transmitting SARS-CoV-2 but are unlikely to be tested.

## INTRODUCTION

Since the start of the coronavirus disease 2019 (COVID-19) pandemic, public health officials worldwide have investigated methods to detect community transmission of severe acute respiratory syndrome coronavirus 2 (SARS-CoV-2). One such method includes environmental surveillance of wastewater, or wastewater-based epidemiology (WBE), that has been used for poliovirus monitoring (1), illicit drug monitoring (2), and detection of antimicrobial resistance (3, 4), among others. The observation that SARS-CoV-2 is shed in human stool (5–8) has led to renewed interest in WBE for COVID-19 surveillance and outbreak monitoring (9–12). Detection of SARS-CoV-2 in wastewater is an early indicator of community transmission trends, often preceding clinical signals, such as new cases or hospitalizations, by 2 to 7 days (13–15). When combined with clinical data, SARS-CoV-2 prevalence in wastewater provides valuable insight to guide public health action. An additional advantage of WBE is that it is an equitable application of public health resources throughout a given community: it captures individuals who would be unlikely to present for clinical testing due to socioeconomic barriers or distrust of the medical community. Finally, WBE has also been used to monitor for potential outbreaks in high-risk institutional settings, such as long-term care facilities, correctional facilities, and dormitories (16–19).

Despite these advantages, WBE testing typically requires a large laboratory due to the need for multiple types of instruments and equipment. Samples are often processed using lengthy and complicated protocols for concentration, extraction, and molecular testing that require specially trained individuals. Sample transport and shipping to centralized laboratories also causes delays, adding additional hours to days of sample processing time, negating its benefit as an early warning system (20). Together, this has created barriers for WBE in remote and isolated settings that lack the resources to perform traditional testing.

To date, no field-deployable and rapid test device for SARS-CoV-2 in wastewater has been developed. Although a preprint article describes a rapid wastewater test from LuminUltra (21), this technology is not automated, requires basic laboratory skills, and currently has limited sensitivity data in real-world situations. The use of lateral flow immunological assays for SARS-CoV-2 in wastewater has been proposed (12, 22, 23); however, in clinical settings, these assays display a low sensitivity compared to the gold-standard nucleic acid amplification tests (24, 25). Additionally, recent work using qualitative loop-mediated isothermal amplification (LAMP)-based assays has shown promise and may provide a fast and affordable option for presence/absence detection of SARS-CoV-2 in wastewater (26).

A possible solution for rapid wastewater testing is the Cepheid GeneXpert system, which supports rapid, fully automated, cartridge-based clinical testing. Recently, Cepheid released a rapid diagnostic multiplex test with a run time of 37 min (27), the Xpert Xpress-SARS-CoV-2/Flu/RSV combination test, for the detection of SARS-CoV-2, influenza A, influenza B, and respiratory syncytial virus (RSV). This assay performs reverse transcription-quantitative PCR (RT-qPCR) targeting the envelope (E) and nucleocapsid (N2) regions of the SARS-CoV-2 genome.

The GeneXpert has several characteristics that make it an ideal candidate for SARS-CoV-2 detection in wastewater compared to other rapid diagnostic tests. The assay’s extraction step uses a filtration system that isolates and concentrates viral particles while removing many of the inhibitors often present in wastewater. Moreover, the assay is one of the most sensitive rapid tests available, with a reported limit of detection of below 50 copies (cp)/mL in clinical settings (27–30). The limit of detection of the GeneXpert can be further enhanced by monitoring the assay’s endpoint fluorescence, a practice used to improve sensitivity in clinical settings when performing high-multiplex sample pooling. Finally, the test is quantitative and provides a cycle threshold (*C_T_*) value that, through the use of a standard curve, can estimate the SARS-CoV-2 concentration in the sample.

To better track and anticipate COVID-19 disease trends, there is a need for an easy-to-use, mobile, and rapid wastewater test for SARS-CoV-2, particularly in remote communities or in resource-limited settings. Consequently, this study aimed to explore the use of the GeneXpert as a solution for SARS-CoV-2 testing in wastewater, which would allow for the decentralization of testing to sampling sites and the capacity to generate near-real-time data to better guide public health actions.

## RESULTS

### Detection of gamma-irradiated SARS-CoV-2 culture in wastewater.

To determine if the GeneXpert SARS-CoV-2 test was compatible with wastewater, gamma-irradiated SARS-CoV-2 was serially diluted in wastewater that was negative for SARS-CoV-2 as determined by the laboratory-developed wastewater test of the Public Health Agency of Canada National Microbiology Laboratory (PHAC-NML) Winnipeg (see Materials and Methods). At input concentrations of 32, 64, and 128 cp/mL, the assay detected SARS-CoV-2 in all replicates (Fig. S1 in the supplemental material). All replicates were negative at input concentrations of 16 cp/mL.

### SARS-CoV-2 detection is robust in unconcentrated wastewater samples from Canadian wastewater treatment plants.

**FIG 1:**
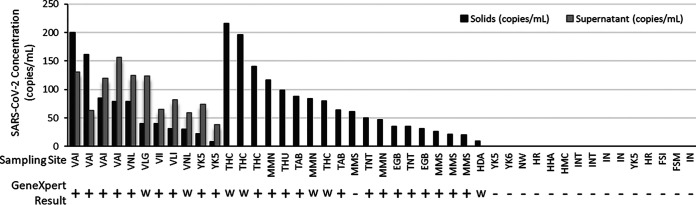
SARS-CoV-2 concentration in Canadian wastewater samples and GeneXpert rapid test results. SARS-CoV-2 concentration was determined using a laboratory-developed, solids-based extraction and RT-qPCR test targeting N1 and N2. Value shown is the average of N1 and N2 targets. For a subset of samples, SARS-CoV-2 concentration was measured in the liquid fraction with a laboratory-developed test. Locations listed multiple times refer to unique samples collected on different days. All 15 samples on the far right of the graph (negative for SARS-CoV-2) were collected from communities with no active SARS-CoV-2 cases. GeneXpert results are either positive (+), weakly positive as determined by endpoint fluorescence (w), or negative (−). EGB, Edmonton Goldbar; HAD, Halifax Dartmouth; HHA, Halifax Halifax; HMC, Halifax Millcove; MMN, Montreal North; MMS, Montreal South; TAB, Toronto Ashbridges Bay; THC, Toronto Highland Creek; THU, Toronto Humber; TNT, Toronto North Toronto; VAI, Vancouver Annacis Island; VII, Vancouver Iona Island; VLG, Vancouver Lions Gate; VLI, Vancouver Lulu Island; VNL, Vancouver Northwest Langley; YK5, Yellowknife lift station 5; YK6, Yellowknife lift station 6; HR, Hay River; FSM, Fort Smith; FSI, Fort Simpson; IN, Inuvik; INT, institutional sample; NW, Norman Wells sewer.

The GeneXpert was used to test wastewater collected in February and March of 2021 from various Canadian communities (method A in Materials and Methods). In total, 30 samples were collected from communities with active COVID-19 cases, and 15 samples were collected from communities with no known COVID-19 activity (Fig. 1). The identities of the specimens were censored until test results were collated. One sample produced a loading error on the GeneXpert system, possibly due to clogging of the test cartridge; however, a repeat test was performed successfully. All samples were tested concurrently with our laboratory-developed test for COVID-19 targeting the solids fraction of wastewater, and for a subset, the supernatant fraction was also tested. Our laboratory-developed tests detected SARS-CoV-2 in all 30 wastewater samples taken from communities with active cases of COVID-19 (Fig. 1). The viral concentration ranged from 9.2 to 216 cp/mL (solids fraction). Of these positives, 22 were reported as positive on the GeneXpert, 7 were endpoint positive (weak positive), and 1 was negative for an agreement of 96.6%. As expected, our laboratory-developed test did not detect SARS-CoV-2 in any of the wastewater samples collected from communities with no active cases of COVID-19 (*n* = 15). The GeneXpert assay also reported all of these samples as negative for SARS-CoV-2 (negative agreement of 100%).

### Wastewater concentration increases GeneXpert test sensitivity in communities with a low prevalence of SARS-CoV-2.

**FIG 2:**
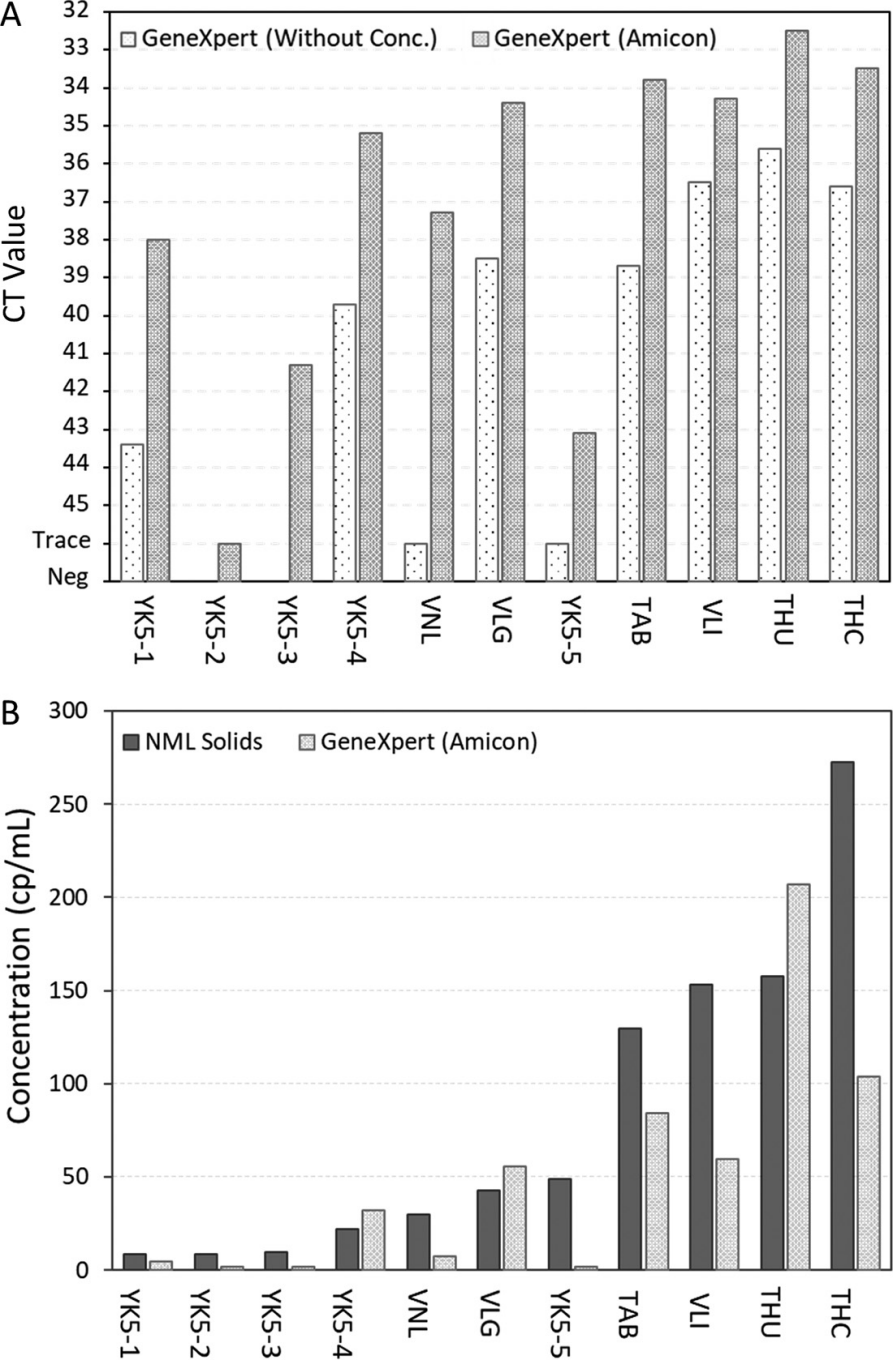
Use of Amicon centrifugal filters to increase sensitivity of the GeneXpert SARS-CoV-2/Flu/RSV assay. (A) The effect of centrifugal filtration on GeneXpert *C_T_* values. Left white bars show *C_T_* values without the use of centrifugal filters; right gray bars show values with the use of centrifugal filters. (B) Quantification of SARS-CoV-2 concentration in wastewater using centrifugal filters and GeneXpert standard curve (light gray) compared to results from laboratory-developed solids assay (dark gray). TAB, Toronto Ashbridges Bay; THC, Toronto Highland Creek; THU, Toronto Humber; VLG, Vancouver Lions Gate; VLI, Vancouver Lulu Island; VNL, Vancouver Northwest Langley; YK5, Yellowknife lift station 5 (multiple time points).

The concentration of samples via Amicon centrifugal filters (method B in Materials and Methods) was used to improve the sensitivity of the GeneXpert rapid test and to investigate if this would allow the test to be used quantitatively (without concentration, the assay’s input of 300 μL makes quantification unreliable due to sampling biases and the heterogeneous nature of wastewater). Eleven wastewater samples were selected from the PHAC Wastewater Surveillance Program with concentrations between 8.5 to 273 cp/mL and tested on the GeneXpert system with or without concentration via centrifugal filtration. Two wastewater samples (YK5-2, YK5-3) were only positive for SARS-CoV-2 when samples were concentrated with centrifugal filters (Fig. 2A). Overall, *C_T_* value decreased by an average of 3.9 cycles, or approximately 16-fold, with the use of centrifugal filters. Following concentration via centrifugal filtration, one sample from a major urban center was also reported as positive for influenza A (data not shown).

A standard curve was developed to convert *C_T_* values into viral load in previous work using the GeneXpert for clinical testing (27, 29). This same curve was used here to convert GeneXpert *C_T_* values into SARS-CoV-2 concentration in wastewater. GeneXpert SARS-CoV-2 concentration was then compared to estimates from the PHAC-NML laboratory-developed solids assay (Fig. 2B). There was a moderate/strong correlation (*r* = 0.724) between the predicted SARS-CoV-2 concentrations of the GeneXpert and the laboratory-developed solids assay (Fig. 2B).

### The GeneXpert SARS-CoV-2 assay can serve as an early warning system for COVID-19 in remote communities without known cases.

**FIG 3:**
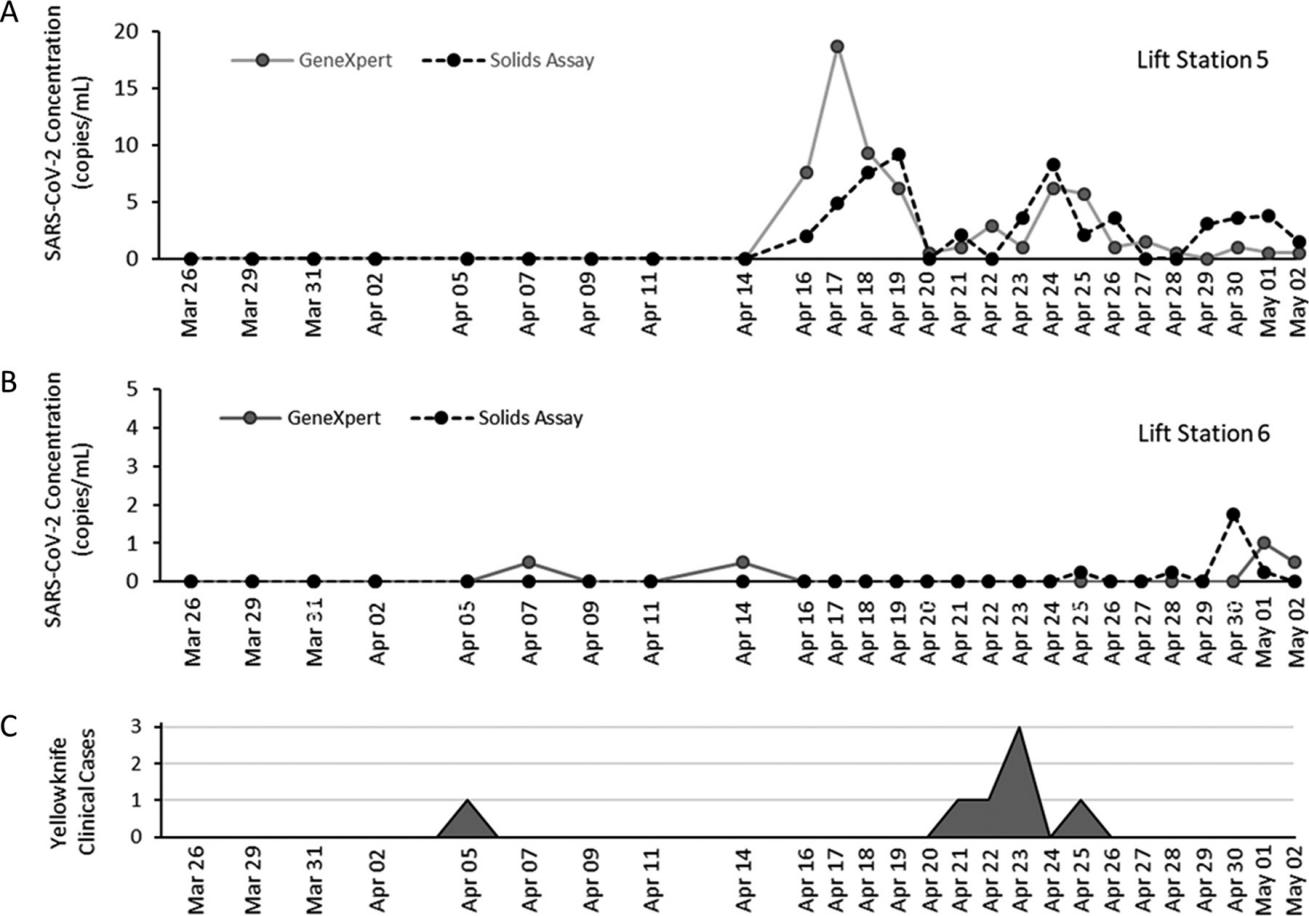
Wastewater surveillance of two Yellowknife lift stations with the GeneXpert and laboratory-developed solids assay. (A) Measured SARS-CoV-2 concentration in lift station 5 wastewater using the GeneXpert-Amicon rapid test (solid gray line) or laboratory-developed solids assay at the National Microbiology Laboratory (dotted black line). Collection frequency increased after first detection. (B) Measured SARS-CoV-2 concentration in wastewater collected at lift station 6. (C) New cases identified in Yellowknife between 26 March and 2 May. One travel-related case was identified on 6 April, and a second independent cluster was identified on 21 to 25 April.

The Public Health Agency of Canada National Wastewater Surveillance Program has been testing wastewater in the Northwest Territories since August 2020. Wastewater samples were shipped from the Northwest Territories to PHAC-NML in Winnipeg for testing with a transit time of 1 to 4 days, with a further 2 days required for testing and reporting. In March 2021, a GeneXpert system was deployed to Yellowknife to support an on-site wastewater surveillance program and expedite test reporting. GeneXpert surveillance in Yellowknife formally began on 26 March with wastewater tested multiple times weekly (Fig. 3).

**FIG 4:**
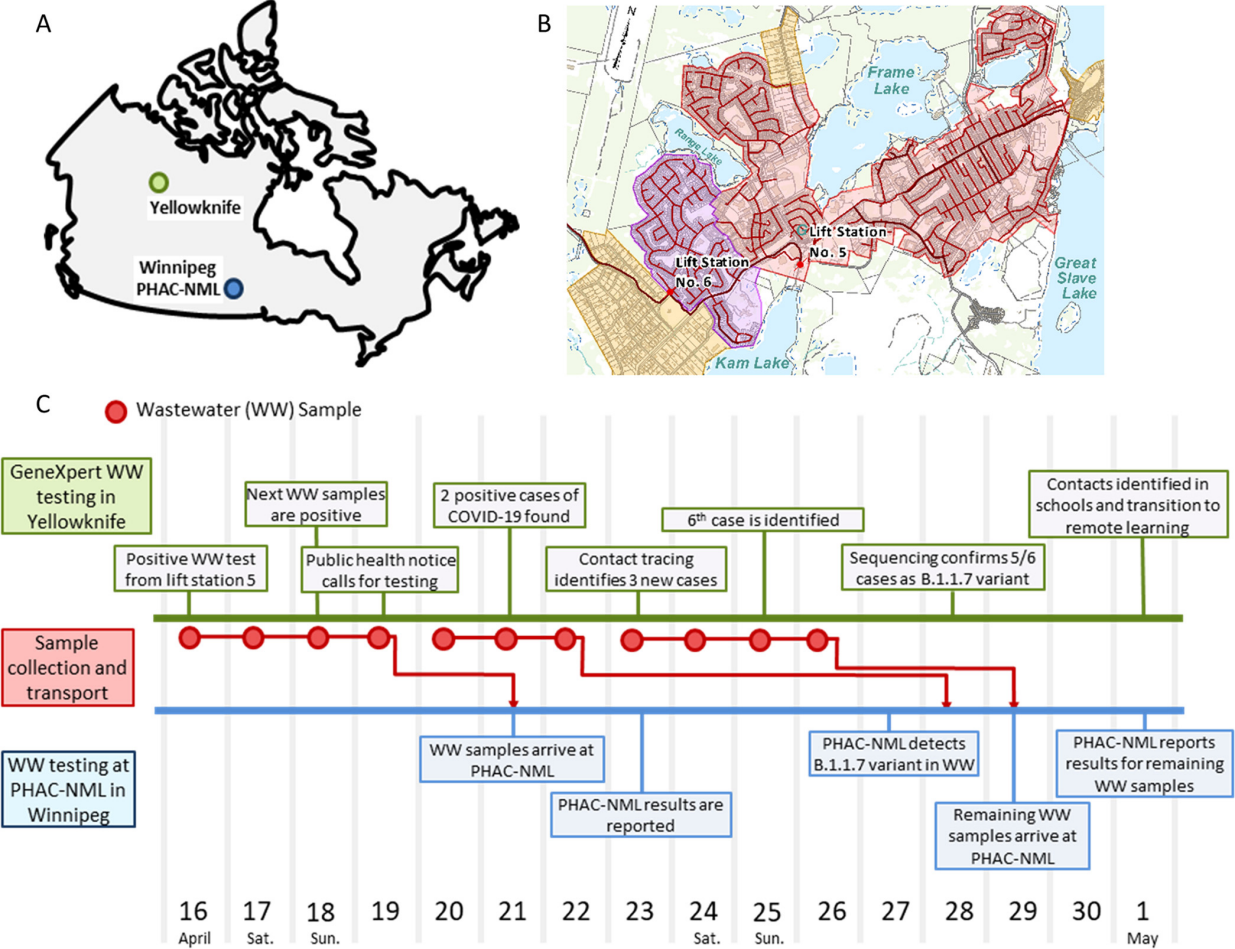
Timeline of events during the GeneXpert wastewater surveillance pilot in Yellowknife, Canada. (A) Map showing locations of Yellowknife (green) and PHAC-NML laboratory in Winnipeg (blue). (B) Two main lift stations (no. 5 and no. 6) in Yellowknife and their corresponding catchments covering >85% of the Yellowknife population. (C) Timeline of events leading to the identification of a SARS-CoV-2 outbreak in Yellowknife. Timing of wastewater test results and public health actions in Yellowknife are shown in green, sample collection and transport events are shown in red, and at PHAC-NML testing is shown in blue.

Preceding the GeneXpert pilot study initiated on 26 March, wastewater samples from Yellowknife were shipped to and tested exclusively at the PHAC-NML laboratory in Winnipeg, Canada, a distance of approximately 1,745 kilometers (Fig. 4A). These wastewater samples were collected from two major lift stations that capture wastewater from >85% of the Yellowknife population (Fig. 4B).

Two weak positives (based on endpoint fluorescence) were observed on the GeneXpert system on 7 April and 14 April (Fig. 3B) in lift station 6 coinciding with a travel-related case that was reported on 5 April (Fig. 3C). Verbal communication with Northwest Territories’ Office of the Chief Public Health Officer acknowledged the wastewater signal from lift station 6 was consistent with information from clinical investigations.

Beginning with samples collected from lift station 5 on 16 April, both the GeneXpert and, subsequently, also the PHAC-NML assay, detected multiple SARS-CoV-2 signals in Yellowknife wastewater. As a response to the first detection of SARS-CoV-2, wastewater collection in Yellowknife was adjusted to daily sampling. Three weeks after initiating the GeneXpert pilot, a persistent SARS-CoV-2 signal was detected in wastewater. Four consecutive positive wastewater detections on the GeneXpert system triggered the Office of the Chief Public Health Officer to initiate a response, including recommending COVID-19 testing for recent travelers to the Northwest Territories (NWT). This testing and subsequent contact tracing led to the identification of a cluster of six cases in the community during the period of 20 to 26 April (Fig. 3C). The timeline for this response is summarized in Fig. 4C.

### Yellowknife GeneXpert wastewater surveillance pilot.

Yellowknife wastewater samples were also shipped to PHAC-NML for confirmatory testing. The first batch of wastewater samples was received by PHAC-NML on 21 April; however, results were reported on 23 April, several days after the first Yellowknife COVID-19 cases had been already identified. Results from the PHAC-NML assay were consistent with the data produced by GeneXpert. As part of the laboratory-developed assay, information is also provided on the variants present in the wastewater sample (31). PHAC-NML reported B.1.1.7 (alpha variant) as the majority variant present in Yellowknife wastewater. This was consistent with clinical sequencing data, which identified most cases in the cluster as B.1.1.7.

## DISCUSSION

Currently, no options exist for mobile and rapid testing of SARS-CoV-2 in wastewater. This study explored the GeneXpert system as a candidate technology for this purpose and performed a pilot study in Yellowknife, Canada. The data presented here demonstrate that the GeneXpert SARS-CoV-2/Flu/RSV assay can reliably detect SARS-CoV-2 in wastewater at concentrations above 32 cp/mL (Fig. S1 in the supplemental material; Fig. 1); however, without concentration, results should primarily be considered qualitative. The sensitivity of the assay can be improved further through concentration methods such as centrifugal filtration, which facilitated detection of SARS-CoV-2 below 10 cp/mL (Fig. 2B). Although effective, concentration by centrifugal filters is not ideal, as it requires the use of a centrifuge, expensive specialized filters, and additional processing time by the operator. Future work should investigate more rapid and deployable methods for concentration, such as filter syringes or concentrating pipettes (32). Alternatively, concentration of the sample may not be required if the GeneXpert system is used to monitor wastewater from a smaller system (small neighborhood or institutional samples).

At the observed level of sensitivity, the GeneXpert is capable of serving as an early detection system in remote communities when paired with a preprocessing method for concentration. This is supported by data from our Yellowknife pilot project, where the GeneXpert system detected SARS-CoV-2 in wastewater before public health officials were aware of its presence in the community. Ultimately, the data produced by the deployed GeneXpert system triggered a testing initiative identifying new COVID-19 cases in the community. Previously, Yellowknife relied exclusively on wastewater testing at a Winnipeg-based testing facility (PHAC-NML), which delayed results by approximately 4 to 7 days due to shipping and sample processing time. In the time between sample collection in Yellowknife and completion of testing in Winnipeg, seven positive wastewater samples were recorded on the GeneXpert system, and five COVID-19 cases were already identified in the community. Since this pilot, the government of Northwest Territories has further expanded GeneXpert testing to include seven additional remote communities. Together, this clearly demonstrates the utility of rapid, deployable wastewater testing for SARS-CoV-2.

In terms of efficient resource allocation, a single GeneXpert cartridge can effectively screen an entire community with much broader coverage than a single clinical test and is likely a more sustainable and cost-effective option for community surveillance in postvaccination scenarios. Additionally, because of the already widespread distribution GeneXpert systems for clinical testing, this technology provides a means to provide immediate global access to wastewater surveillance, including remote communities and low-income countries. In particular, this can make an impact in low-income countries where GeneXpert-based surveillance systems are already in place for tuberculosis, HIV, and COVID-19 testing (33–38). Although the use of an autosampler for sample collection is currently preferred for GeneXpert testing, other low-cost options, such as passive samplers, are currently being explored. Additionally, wastewater sample collection in remote communities can be challenging, particularly in communities with nonsewered systems. Innovative solutions, such as passive samplers (39) or collection systems on sewage trucks, provide possible areas of focus to improve access to testing in remote communities.

There are some limitations to the GeneXpert technology. Notably, the targets are determined by the manufacturer, and the system itself is optimized for clinical testing. The system currently does not monitor for SARS-CoV-2 variant detection nor include fecal indicators, such as pepper mild mottle virus, that may be useful for data normalization (40). Additionally, extraction efficiency cannot be measured through the use of recovery controls, as assay targets are predefined by the manufacturer. As the cartridge uses an internal filter, there is also a risk of clogging; however, this was only observed once in this study, and the sample was successfully processed in a repeat test. Although we only observed one instance of a cartridge clogging in this study, it could potentially be an issue in sewer sheds with a high content of suspended solids. The input volume of the GeneXpert SARS-CoV-2/Flu/RSV test is also quite low at 300 μL, which may introduce sampling biases when testing large volumes of heterogeneous wastewater. This sampling bias makes any quantitative data from the GeneXpert less reliable unless a concentration step is performed. Ideally, wastewater-specific test cartridges for the GeneXpert could be developed with optimized targets and larger input volumes, or a similar large-volume assay could be developed using a competing technology.

The Xpert Xpress SARS-CoV-2/Flu/RSV assay used in this study is also used clinically for the detection of influenza A, influenza B, and respiratory syncytial virus. Therefore, it is theoretically possible that this assay will be able to detect these other pathogens in wastewater in addition to SARS-CoV-2. In this study, we only observed one positive detection of influenza A in a wastewater sample from a major Canadian city and no detection of influenza B or respiratory syncytial virus. The lack of detection may be reflective of the low transmission rates of influenza and other respiratory viruses from 2020 to 2021 (41–43), or it may indicate that the GeneXpert is not suitable for surveillance of these pathogens, warranting further studies. Other clinical tests for the GeneXpert system have targets likely to be present in wastewater such as norovirus, Clostridium difficile, and various antimicrobial resistance genes. Of particular importance in remote and isolated communities would be surveillance for active tuberculosis (44, 45). It would also be of interest to investigate if these clinical GeneXpert assays are also compatible with wastewater as an input.

Taken together, this work highlights the importance of rapid wastewater testing for SARS-CoV-2. Although previous studies have shown wastewater signals may provide several days of lead time compared to clinical cues of transmission, much of this early warning is eroded by delays resulting from sample shipping and processing times. Centralized laboratories in urban centers often provide wastewater surveillance for remote and isolated communities; however, these locations are often separated by large distances, creating logistical complications. As the second-largest country by land mass, wastewater tests will need to be deployable for an effective and equitable Canadian national wastewater surveillance program for COVID-19. Additionally, WBE has not been substantially applied to low-resource communities (46). Vaccinations and effective clinical surveillance have not reached many parts of the developing world, and these factors increase the risk and impact from the introduction of COVID-19 into these communities (https://ourworldindata.org/coronavirus). Although not specifically designed for this purpose, the GeneXpert system provides a stopgap measure until a purposely designed wastewater rapid test for SARS-CoV-2 becomes available for remote and low-resource communities. Ideally, future solutions should provide real-time or near-real-time monitoring of SARS-CoV-2 and other pathogens in wastewater to inform public health responses.

## MATERIALS AND METHODS

### Wastewater sample collection.

Primary influent or raw wastewater samples were collected from 2 institutions and 25 metropolitan and remote wastewater collections systems across Canada. Metropolitan sites included Toronto, Montreal, Vancouver, Edmonton, and Halifax; all samples from these sites were collected from mechanical wastewater treatment plants. Remote sites included five communities in the Northwest Territories, Yellowknife, Hay River, Inuvik, Fort Smith, and Fort Simpson. All samples from remote sites were collected from lift stations. Finally, two institutional samples were included in this work; each site houses between 300 and 800 long-term residents. All samples were composites collected in fresh polyethylene terephthalate bottles. All samples for this study were collected between February and May 2021. Samples that were transported for testing to PHAC-NML were shipped on ice packs and, once received, were refrigerated at 4°C for up to 48 h until testing was performed.

Yellowknife lift station 5 and lift station 6 were selected for our pilot study. Lift station 5 serves approximately 18,200 residents, with an average daily flow rate of 6,683 m^3^. Lift station 6 serves approximately 3,200 residents, with an average daily flow rate of 1,196 m^3^. Lift station 5 has a mix of residential, commercial, and industrial properties within the catchment, whereas lift station 6 covers primarily residential properties. Yellowknife does not have a combined storm and sanitary sewer system.

### Preparation of wastewater samples for GeneXpert testing.

Two methods were used to prepare wastewater samples for processing on the GeneXpert system. These methods are referred to as method A (no concentration) and method B (concentration of supernatant via Amicon centrifugal filtration).

### (i) Method A.

We added 15 μL of 10% Tween 80 (final 0.01% [vol/vol]) to 15 mL of untreated wastewater. The sample was then vortexed at maximum speed for 20 s and then rested for 2 min to allow debris to settle. Once settled, 300 μL of sample was loaded directly into the GeneXpert cartridge and analyzed as per the manufacturer’s protocol for clinical specimens.

### (ii) Method B.

We added 15 μL of 10% Tween 80 (final 0.01% [vol/vol]) to 15 mL of wastewater, and it was vortexed at maximum speed for 20 s. The wastewater was then clarified via centrifugation for 20 min at 4,200 × *g* and 4°C. The supernatant was decanted into an Amicon Ultra15 10-kDa centrifugal filter unit (MilliporeSigma, Burlington, MA), and care was taken to avoid dislodging of the pellet. The Amicon filtration devices were centrifuged at 4,200 × *g* for 30 min and 4°C. Molecular-grade water was added to concentrates to bring the volume to 300 μL (input volume for GeneXpert testing). Next, the sample was loaded directly into the GeneXpert cartridge and analyzed as per the manufacturer’s protocol for clinical specimens. The Pearson correlation coefficient was used to compare the SARS-CoV-2 concentration as measured by the GeneXpert system and Amicon filtration to traditional laboratory-developed qPCR tests.

### Mobile wastewater testing for SARS-CoV-2 with the GeneXpert system.

All wastewater samples were tested on the GeneXpert XVI (Cepheid, Sunnyvale, CA) using the Xpert Xpress-SARS-CoV-2/Flu/RSV cartridge (Cepheid, Sunnyvale, CA). GeneXpert tests were performed at PHAC-NML in Winnipeg, Canada, and the Taiga Environmental Laboratory in Yellowknife, Canada. The endpoint fluorescence was also monitored for each run, where an endpoint fluorescence of >10 is indicative of a weakly positive sample (reported to public health as a trace detection). The use of GeneXpert endpoint fluorescence to capture weak positives is a practice used in some Canadian hospitals to determine if pooled samples should be split for individual testing. For concentrated wastewater samples (method B), SARS-CoV-2 concentration was determined using a standard curve developed in our previous work (27, 29). To conserve GeneXpert supplies for clinical testing, all initial experiments were performed with unused cartridges from open test kits that were sampled as part of lot quality management.

### Preparation of wastewater nucleic acid extracts for conventional laboratory testing.

PHAC-NML has protocols to process both the supernatant and solid fractions of wastewater (31). Samples received from Northwest Territories and Vancouver underwent both the supernatant and solids processing methods, while only the solids were processed for samples received from Toronto, Montreal, Edmonton, and Halifax wastewater treatment plants as per the preferences of these communities.

The method used to process the supernatant followed the same concentration steps outlined in method B, with the exception of a filtration centrifugation time of 35 min. After filtration, 2 mg of carrier RNA (Sigma-Aldrich, St. Louis, MO) and 700 μL of MagNA Pure 96 external lysis buffer (Roche, Pleasanton, CA) was added to the processed supernatant before extraction on the MagNA Pure 96 instrument (Roche, Pleasanton, CA).

For processing the solids fraction, 30 mL of wastewater was centrifuged for 20 min at 4,200 × *g* and 4°C. After centrifugation, the supernatant was carefully removed, and the pellet was transferred to a bead-beating tube containing 200 μL of 0.5-mm zirconia/silica beads and 700 μL of RLT lysis buffer with 1% β-mercaptoethanol (Qiagen, Germantown, MD). Samples were homogenized using a Bead Mill 24 homogenizer (Fisher Scientific, Hampton, NH) for 4 cycles of 30 s at 6 m/s with 20 s of rest time between each cycle. Subsequently, the homogenate was clarified at 12,000 × *g* for 3 min, and 1,000 μL of the clarified homogenate was transferred to a 96-well MagNA Pure 96 processing cartridge containing 2 mg of carrier RNA. The extraction was then performed on the MagNA Pure 96 instrument (Roche, Pleasanton, CA).

### Laboratory-based wastewater testing for SARS-CoV-2 by RT-qPCR.

SARS-CoV-2 was quantified in 5 μL of lysate using the TaqPath 1-Step RT-qPCR master mix, CG (Life Technologies, Carlsbad, CA) with N1 and N2 primer and probe sequences obtained from the U.S. Centers for Disease Control and Prevention (Table S1 in the supplemental material) (https://www.cdc.gov/coronavirus/2019-ncov/lab/rt-pcr-panel-primer-probes.html). The RT-qPCR was prepared as per the manufacturer’s instructions with a primer concentration of 500 nM and probe concentration of 125 nM. The following cycling conditions were used: 25°C for 2 min, 50°C for 15 min, and 95°C for 2 min, followed by 40 cycles of 95°C for 5 s and 60°C for 30 s. RT-qPCR was performed with two technical replicates. Quantification of the SARS-CoV-2 N1 and N2 *C_T_* values was performed using a 5-point standard curve prepared from synthetic DNA oligonucleotides. All TaqMan-based RT-qPCR assays were performed on a QuantStudio 5 real-time PCR system (Life Technologies, Carlsbad, CA).

Invitrogen’s GeneArt Strings DNA fragments were used to prepare a standard curve for each of the targets in this study. These controls consist of double-stranded linear DNA fragments assembled from synthetic oligonucleotides. Each standard arrived lyophilized, and upon use, they were reconstituted to 5.00 × 10^9^ genome copies/μL. Immediately after reconstitution, each standard was aliquoted into single-use Lo-Bind plastic tubes and stored at −80°C. DNA standards were single use and were never subjected to more than one freeze-thaw cycle. A five-point standard curve was prepared for each target in triplicate. The standard curve parameters are found in Table S2.

### Standardization of SARS-CoV-2 measurements.

High-titer SARS-CoV-2 culture (strain VIDO; GISAID accession no. EPI_ISL_425177), inactivated by gamma irradiation, was provided by the Special Pathogens Program of PHAC-NML. Briefly, SARS-CoV-2 was cultured by PHAC-NML Special Pathogens in Vero cells in minimum essential media, and cellular debris was removed via low-speed centrifugation. The viral supernatant was inactivated via gamma irradiation using a Gammacell 220 Cobalt-60 irradiator with a total exposure of 3 Mrad of radiation. Inactivated viral culture was quantified using the GeneXpert system and standard curve, as well as through droplet digital PCR.

### Biosafety measures.

All SARS-CoV-2 culturing and subsequent inactivation were performed in a certified containment level 3 laboratory at PHAC-NML. To minimize the risk of large wastewater spills, processing and collection volumes were minimized and kept to below 1 L. Wastewater was processed at PHAC-NML according to protocols established by Safety and Environmental Services, including the use of personal protective equipment, use of a biosafety cabinet, and inactivation of wastewater before disposal. Wastewater was tested at TAIGA environmental laboratory according to their established procedures for wastewater testing, including the use of personal protective equipment, biosafety cabinets, and inactivation of wastewater before disposal. No biosafety incidents occurred during this study.
