# Supplementary material for: A Sensitive and Rapid Wastewater Test for SARS-COV-2 and Its Use for the Early Detection of a Cluster of Cases in a Remote Community
Source: Appl Environ Microbiol. 2022 Mar 8;88(5):e01740-21. doi: 10.1128/aem.01740-21 (PMC8904056; doi:10.1128/aem.01740-21)
Supplement: Supplemental file 1 — Tables S1 and S2 and Fig. S1 and S2. Download aem.01740-21-s0001.pdf, PDF file, 0.5 MB [file aem.01740-21-s0001.pdf]

Supplementary Material:

Supplementary Table 1: List of SARS-CoV-2 Primers and Probes used for the Laboratory-Developed q-RT-PCR Test (Source, US Centers for Disease Control and Prevention).

**Assay**	**Oligonucleotide**	**Sequence (5' -- 3')**	**Concentration (µM)**
N1	2019-nCoV_N1 forward primer	GAC CCC AAA ATC AGC GAA AT	0.5
	2019-nCoV_N1 reverse primer	TCT GGT TAC TGC CAG TTG AAT CTG	0.5
	2019-nCoV_N1 reverse probe	FAM-ACC CCG CAT TAC GTT TGG TGG ACC-NFQ	0.125
N2	2019-nCoV_N2 forward primer	TTA CAA ACA TTG GCC GCA AA	0.5
	2019-nCoV_N2 reverse primer	GCG CGA CAT TCC GAA GAA	0.5
	2019-nCoV_N2 reverse probe	FAM-ACA ATT TGC CCC CAG CGC TTC AG-NFQ	0.125

Supplementary Table 2: Standard Curve Parameters for the N1 and N2 primers used for the laboratory-developed wastewater test in this study.

	**N1**	**N2**
**Slope**	-3.36	-3.43
**Intercept**	36.27	37.02
**R^2**	0.9979	0.9965
**Efficiency**	98.44	95.68

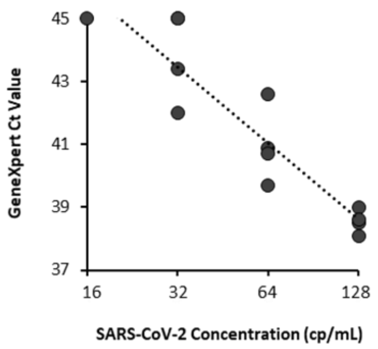

Input Concentration (cp/mL)	GeneXpert CT value			
	Replicate # 1	Replicate #2	Replicate #3	Replicate #4
128	38.5	38.1	39.0	38.6
64	39.7	40.9	40.7	42.6
32	42	43.3	Endpoint Pos.	Endpoint Pos.
16	Negative	Negative	Endpoint Pos.	Negative

Supplementary Figure 1: Detection of SARS-CoV-2 in wastewater using the GeneXpert system. Gamma irradiated SARS-CoV-2 culture fluid was diluted in wastewater to final concentrations between 16 – 128 copies (cp/mL). All points in the dilution series were tested using four independent replicates, diluted in negative wastewater samples collected from four locations across Canada. All inputs of greater than 32 cp/mL were identified as positive or endpoint positive (pos.) on the GeneXpert system.

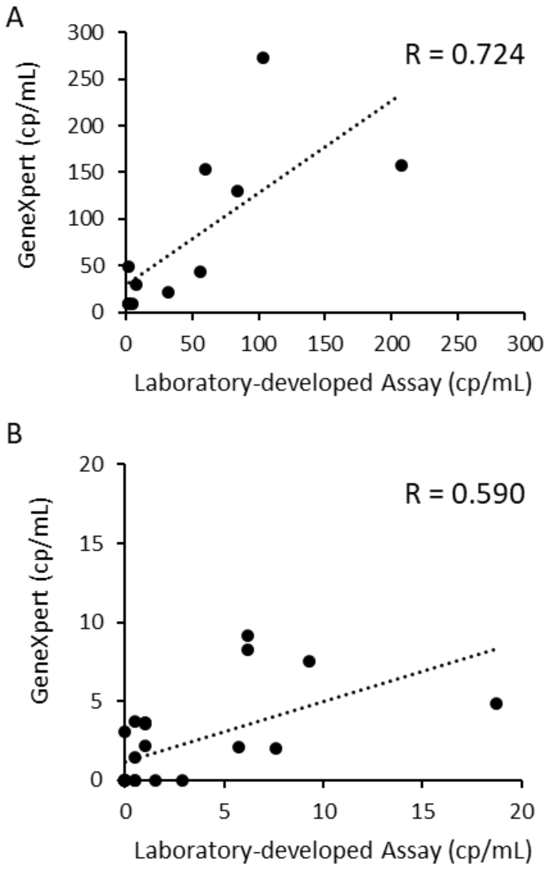

Supplementary Figure 2: Correlation of GeneXpert (with Amicon concentration) and laboratory-developed assay for the quantification of SARS-CoV-2 in wastewater. (A) Correlation of SARS-CoV-2 quantification with the GeneXpert and laboratory-developed assay using input wastewater samples collected from multiple sites across Canada (See Fig 2B). (B) Correlation of SARS-CoV-2 quantification with the GeneXpert and laboratory-developed assay using input wastewater samples collected from Yellowknife Lift Station 5 (See Fig 3A).
